# Operationalizing Principle-Based Standards for Animal Welfare—Indicators for Climate Problems in Pig Houses

**DOI:** 10.3390/ani8040044

**Published:** 2018-03-23

**Authors:** Herman M. Vermeer, Hans Hopster

**Affiliations:** Wageningen Livestock Research, P.O. Box 338, Wageningen 6700 AH, The Netherlands; hans.hopster@wur.nl

**Keywords:** pigs, climate, principle-based standards, principle-based regulation, inspection, welfare, welfare regulations, animal-based indicators

## Abstract

**Simple Summary:**

Dutch farms that probably do not comply with the legal principle-based standard for climate in pig houses can be identified based on a limited set of measurements. The results may encourage pig farmers to improve climatic conditions, but can also justify subsequent investigation to substantiate noncompliance with the legal animal welfare standards. This was concluded after farm data collection by inspectors on 96 farms with weaners or growing–finishing pigs. Analysis of the data revealed that CO_2_ and NH_3_ concentrations; pig fouling; and ear, tail, and eye scores can be used as indicators of suboptimal climatic conditions.

**Abstract:**

The Dutch animal welfare law includes so-called principle-based standards. This means that the objective is described in abstract terms, enabling farmers to comply with the law in their own way. Principle-based standards are, however, difficult for the inspection agency to enforce because strict limits are missing. This pilot project aimed at developing indicators (measurements) to assess the climate in pig houses, thus enabling the enforcement of principle-based standards. In total, 64 farms with weaners and 32 farms with growing–finishing pigs were visited. On each farm, a set of climate-related measurements was collected in six pens. For each of these measurements, a threshold value was set, and exceeding this threshold indicated a welfare risk. Farm inspections were carried out during winter and spring, thus excluding situations with heat stress. Assessment of the variation and correlation between measurements reduced the dataset from 39 to 12 measurements. Using a principal components analysis helped to select five major measurements as warning signals. The number of exceeded thresholds per pen and per farm was calculated for both the large (12) and small (five) sets of measurements. CO_2_ and NH_3_ concentrations were related to the outside temperature. On colder days, there was less ventilation, and thus CO_2_ and NH_3_ concentrations increased. Air quality, reflected in the CO_2_ and NH_3_ concentrations, was associated with respiratory problems. Eye scores were positively correlated with both pig and pen fouling, and pig and pen fouling were closely related. We selected five signal indicators: CO_2_, NH_3_, and tail and eye score for weaners and finishers, and added ear score for weaners and pig fouling for growing–finishing pigs. The results indicate that pig farms can be ranked based on five signal indicators related to reduced animal welfare caused by climatic conditions. This approach could be adopted to other principle-based standards for pigs as well as for other species.

## 1. Introduction

The Dutch Animal Welfare legislation contains principle-based standards. The objective is described in abstract terms, enabling farmers to comply with the law in their own way. This means that the welfare goal of the principle-based standard is formulated in abstract terms. Thus, within the framework of the legislation, the pig farmer may choose his own route to achieve that goal. However, the enforcement of principle-based standards is difficult for the inspection agency because strict limits are missing. Inspection with rule-based regulations is easier than with principle-based regulations for the legal protection of animal welfare [1]. Principle-based standards challenge the inspection agency and the pig farmers to develop new routes to guarantee animal welfare other than rule-based regulation. In addition, new inspection tools must be developed to check whether the legal goal has been achieved by the pig farmer. Welfare assessment requires specific expertise and training in observing animal behaviour. Generally, there is a need for significant alerts that could be used as a starting point for further investigation of the extent to which the pig farmers comply with the legal standards in practice.

The principle-based standard for climatic conditions, as outlined in the Dutch law for animal owners (Besluit Houders van Dieren) [2], is that air circulation, air content, temperature, relative humidity, and gas concentrations around the animal should not be harmful to the animal. This is a good example of a principle-based standard. By providing a good climate in pig houses, an environment will be created in which the animals feel well and stay healthy. The inspection of the indoor climate therefore should include controlling the various indoor climate properties, namely air circulation, air content, temperature, relative humidity, and gas concentrations, in relation to animal welfare, as reflected in animal-based indicators. For pigs, there are no legal threshold values for these characteristics. For poultry, however, values up to a maximum of 3000 ppm CO_2_ and 20 ppm NH_3_ are legally permitted in the Netherlands [2] and Germany [3]. Employees should not be exposed for more than 8 hr at 20 ppm NH_3_ or 15 min at 50 ppm NH_3_ or more [4]. In the German pig welfare law [3], an upper limit of 20 ppm is stated for the NH_3_ concentration. It is therefore reasonable to use 20 ppm as the limit for the concentration of NH_3_ in a pig house. Despite the lack of legal limits for CO_2_ and NH_3_ in Dutch pig houses, the principle-based standard for the indoor climate should be complied with and maintained. Animal-based indicators, in particular, give insight into the degree of the reduction of animal welfare. Characteristics that give a first indication that the indoor climate does not meet the norm are referred to as “signal indicators” in this paper. Exceedances of these signal indicators could lead to further investigation, and finally, sanctions. 

In our study, climate-related data obtained in houses for weaners or for growing–finishing pigs were collected by the Dutch national inspection agency (NVWA). In this data, we primarily looked for reliable indicators that signalled climate-related welfare problems. In addition, we looked for consistency between measurements, with the aim of reducing the number of parameters and thereby increasing the workability of the protocol for inspectors. With this set of signal indicators, the aim was to facilitate the enforcement of the principle-based standard for climatic conditions for weaners and growing–finishing pigs.

The indoor air quality can be assessed through NH_3_ and CO_2_ concentrations. Jones et al. [5] gave pigs the choice to stay in environments with either 10, 20, 30, or 40 ppm NH_3_. Pigs appeared to spend 80% of their time in environments with 10 or 20 ppm, and avoided higher concentrations. Wathes et al. [6] indicated in a review article that there are no health disorders in pigs held in a room with an NH_3_ concentration of up to 50 ppm. Parker et al. [7] found more aggression and less submissive behaviour in pigs kept at a NH_3_ concentration of 20 ppm, when compared with 5 ppm. 

For CO_2_, no negative effects are known for pigs under commercial conditions, but the limit for humans is 5000 ppm (0.5%) [8]. Raj and Gregory [9] stated that, “most of the pigs did not show aversion to the presence of 30% (300,000 ppm) carbon dioxide in air”. This level is far above the level that will ever be reached under commercial conditions. In this project, CO_2_ is mainly a measure of ventilation rate, which, if substandard, is more harmful than the gas itself at this concentration. 

Pig welfare will not be impaired when the ambient temperature is within their comfort zone [10]. In this comfort zone, pigs hardly have to adjust their behaviour to keep their body temperature constant. Within the wider thermoneutral zone, pigs keep their body temperature constant by adapting their behaviour. At low temperatures (towards the lower critical temperature), pigs increase their core body temperature by lying in close proximity to each other and increasing muscular energy expenditure (shivering). At high temperatures (towards the upper critical temperature), pigs increase heat loss through conduction (e.g., by lying on a wet floor), by avoiding physical contact with conspecifics, and they decrease their feed intake. These behavioural adaptations signal reduced welfare [10]. Also, results of behavioural frustration such as ear and tail biting may point to a poor indoor climate [11,12]. As Fablet et al. described [12], we expected to find relationships between the climatic conditions and animal-based indicators both on-farm and at the slaughterhouse.

The aim of this pilot project was therefore to find indicators that enable the identification of farms that could be suspected of not complying with the principle-based standard for climate in pig houses. Instead of replacing the principle-based standard by rule-based regulations, we aimed to provide Dutch pig farmers and inspectors with a method that would justify further investigation on farms with high scores on signal indicators.

## 2. Materials and Methods

In cooperation with the Dutch national inspection agency (NVWA), we developed a monitoring protocol. Pairs of experienced inspectors visited 96 conventional pig farms, divided into 64 farms with weaners and 32 farms with growing–finishing pigs. The majority of the weaners (in 59 of the 64 farms) were housed on fully slatted plastic floors, without bedding, and half had their tails docked. All growing–finishing pigs were housed on partly slatted concrete floors, without bedding, and had half of their tails docked. Separate datasets for weaners (4–10 weeks of age, 7–25 kg) and for growing–finishing pigs (10–26 weeks of age, 25–120 kg) were created. These datasets each contained 6 records (pens) per farm, with 2 age groups for the weaners and 3 age groups for the growing–finishing pigs. The data were analysed stepwise to narrow down the number of measurements into a workable selection. For the selected measurements, threshold values regarding animal welfare were set based on the literature. Then, for each farm, the number of times they exceeded a threshold value was recorded. The details are described in the following sections.

### 2.1. Data Collection

At the end of 2015, animal scientists and employees of the inspection agency together composed a list of 39 characteristics related to the indoor climate in pig houses. These lists were almost identical for weaners and growing–finishing pigs. Choices were based on available inspection equipment and available time per inspection, combined with the expected overlap between measurements. For example, air replacement, measured as CO_2_ concentration, affects most harmful gases in the indoor environment and has therefore been regarded as a very appropriate characteristic of air replacement. The animal-based indicators were similar to those in the welfare quality pig protocol [10].

The measurements were recorded at pen level, except for the measurements from category 8, “Performance at a farm level”. Data were grouped into the following categories:

1. General information, such as date, farm number, inspector names, pen number, and the reason for selecting the pen (randomly chosen rooms, with the worst pen(s) chosen based on fouling and/or lesions); data were grouped by weaners (3 pens of young weaners (aged 5–7 weeks) and 3 pens of older weaners (aged 8–10 weeks)) and growing–finishing pigs (2 pens for young (aged 11–15 weeks), 2 pens for middle-aged (aged 16–20 weeks) and 2 pens for old pigs (aged 21–25 weeks)).
2. Dimensions of the pen and room, including the number of animals for the calculation of the area and volume per animal, and air inlet system.
3. Indoor and outdoor temperature, and CO_2_ and NH_3_ concentrations at the animal level measured with the MultiRAE II Lite multigas monitor (RAE Systems, Sunnyvale, CA, USA).
4. Pen and animal fouling on a 3-level scale: 0 = 0–20%, 1 = 20–50%, 2 ≥ 50% wet and/or dirty floor/skin.
5. Tail-, ear-, and eye score; the score of the individual pig with the worst score is taken as the pen score following the WQ pig protocol [10]:
  Tail score: 0 = healthy; 1 = small lesions/scratches; 2 = bigger lesions/wounds with fresh blood.
  Ear score: 0 = healthy and intact; 1 = black crusts; 2 = fresh, red lesions.
  Eye score: 0 = white and clean; 1 = red and clean; 2 = white and dirty; 3 = red and dirty.
6. Breathing characteristics:
  Panting: number of pigs panting.
  Pumping: number of pigs pumping.
  Coughing and sneezing: number of pigs coughing and sneezing within 5 min.
7. Lying and activity:
  Huddling: 0 = no huddling; 1 = up to 20% of the pigs in the pen are huddling; 2 = more than 20% of the pigs in the pen are huddling.
  Shivering: 0 = no shivering; 1 = up to 20%; 2 = more than 20% of the pigs are shivering.
  Lying isolated: 0 = lying as a group; 1 = 50% isolated; 2 = more than 50% of the pigs are lying isolated.
  Lying posture: score for majority of group; 0 = sternal; 1 = partly lateral; 2 = lateral.
  Activity: number of standing/sitting pigs per pen when inspectors left the room.
8. Performance on the farm level on a yearly basis:
  Mortality: mortality (%), including euthanasia.
  Medication: daily doses of antibiotics per pig.
  Slaughter information: pneumonia (%) and pleurisy (%), only for growing–finishing pigs.

### 2.2. Data Analysis

Both datasets (64 weaner and 32 growing–finishing farms) were initially summarized at a farm level with all measurements in one dataset. These datasets were analysed for correlations between the different characteristics.

Using a principal components analysis (PCA in Genstat) [13], the data was graphically displayed in biplots. Standardization and normalization of the measurements were performed beforehand. In a biplot, data with many dimensions (due to the inclusion of more than two measurements at a time) makes a two-dimensional image with the maximum explained variance.

The selection of the measurements in the biplot was based on expert knowledge in how to present the most important characteristics. For example, a high outdoor temperature is usually related to a high ventilation rate, resulting in better air quality. Both the ventilation rate and NH_3_ production clearly contributed to the quality of the climate and were therefore included in the basic biplot.

After establishing the basic biplot, we looked at how the other characteristics were related in the graph, one by one. Positive relations between animal-based indicators and environmental measurements (such as CO_2_ and NH_3_ levels) were assessed visually in a biplot prior to testing in a mixed model (regression) analysis of the significance of these relations. By means of cross-table analysis, the relations between (animal) characteristics were tested. In this way, animal-based indicators that correlated well with the axes of ventilation rate and ammonia production were found. We chose the best-fitting indicators, with the other indicator axes in the basic biplot as additional signal indicators.

### 2.3. Selection of Potential Indicators

In order to reduce the extended set of measurements, we took the following steps to reduce unnecessary or unreliable measurements, including:

1. Measurements that were not relevant as indicators for the final analysis, but had an “administrative/log function” (such as farm ID and inspectors).
2. Supporting measurements necessary to calculate a key measurement, but which were not required for the final dataset (such as pen and room dimensions).
3. Measurements that could not be recorded in a reliable way and measurements with missing values or those that were not practically feasible (like activity and mortality).
4. A final step was taken to see whether a small group of “signal indicators” could be selected from the dataset.

### 2.4. Threshold Values

For nine of the animal-directed scores [14], we used the welfare quality (WQ) pig protocol [10], which was established after the consultation of international experts on pig welfare. In this protocol, most measurements are scored in three classes: 0 = good; 1 = warning; 2 = alarm. In this project, the threshold value of 1 has been applied, because this warning value already indicates impaired welfare. Although the eyes were scored in four classes, we used the threshold value of 1, i.e., with dirty and/or red eyes. There were no threshold values available for mortality, antibiotic use, and pleurisy/pneumonia. In these cases, we used the Dutch benchmark (antibiotic usage), the average (slaughter data), or the Dutch legal minimum value (space allowance).

For each farm, we determined the total number of times that the threshold values were exceeded for each of the measurements, as well as the highest (worst) average pen score per farm.

Table 1 gives an overview of the measurements, with threshold values for weaners and growing–finishing pigs. Scores equal to or higher than these threshold values were considered as a signal of reduced animal welfare.

## 3. Results

The farm visits took place in the winter and spring of 2016, with outdoor temperatures between 0 and 27 °C. Eight percent of the total number of observed pens were selected based on signals of suboptimal climate, as noted through coughing (1%), the eye score (1%), the ear score (1%), and fouling (5%). The other pens (92%) were selected at random.

### 3.1. Weaners

The measurements of the weaner pigs, taken at a pen level, are summarized in Table 2.

**Figure 1:**
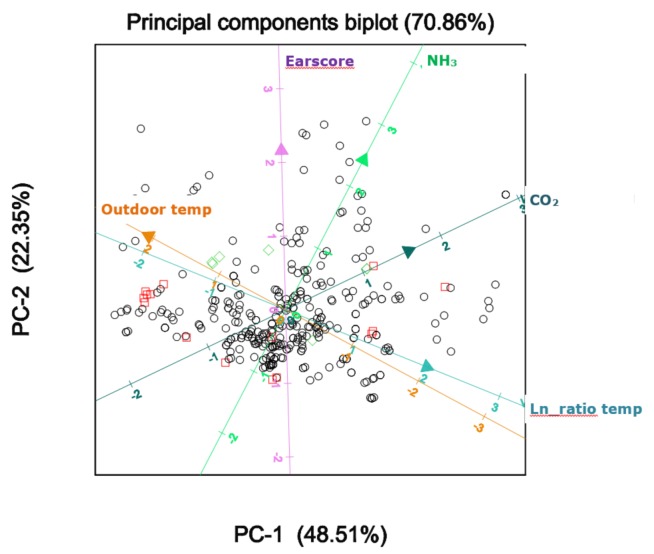
Basic biplot for weaners with outdoor temperature, natural logarithm (ln) of the ratio of indoor:temperature to outdoor temperature, and NH_3_ and CO_2_ levels, with ear score as an additional indicator. PC: principle component.

The principal components analysis (PCA) of the weaner dataset resulted in a basic biplot with CO_2_, NH_3_, outdoor temperature, and the ratio between the outdoor and indoor temperature as a measure of ventilation rate. The axes of the animal-based indicators for ear score, eye score, and tail score pointed in the same direction as that of NH_3_. The PCA results of the behavioural indicators were less clear, except for shivering. Pig and pen fouling were considered irrelevant for weaners, because they were housed on fully slatted floors, which on most of the farms did not lead to fouling problems. Figure 1 shows an example of the basic biplot, with ear score as an additional parameter. Subsequently, the distinct animal-based indicators were tested for their relationship with the basic axes in the biplot. In total, 12 measurements were used in this analysis.

Table 3 shows the results of the relationships between the most important indicators at the farm level. Relationships between the animal-based measurement and NH_3_ level or CO_2_ level were analysed with mixed model regression analysis, and the relationships between animal-based indicators were analysed using cross-table analysis with Genstat [13]. The major results showed that the outdoor temperature had a significant negative relationship with the NH_3_ and CO_2_ level, and that the NH_3_ and CO_2_ level were positively related to each other. The age group of the weaners was not related to any of the measurements. The respiratory traits of panting and pumping were positively related to such an extent that a choice for one of the respiratory traits could be sufficient. The animal-based indicators of tail score and ear score were positively related. No relationship was found between the NH_3_ level and animal-based measurements except shivering; a closer look at the data showed that the outcome for shivering was largely caused by the data from one farm. Finally, shivering, huddling, and posture were positively related to each other.

Based on these analyses, as well as expert knowledge, a set of five measurements was retained as potential “signal indicators”. These were the CO_2_ level, NH_3_ level, ear score, tail score, and eye score. The CO_2_ and NH_3_ levels were both indicators of the air quality and were negatively related to outdoor temperature. Thus, with a higher outdoor temperature, the levels of CO_2_ and NH_3_ were lower. Ear score, eye score, and tail score did not show strong mutual relationships, but each was related to a history of problems with the climatic conditions. Behavioural indicators, such as huddling, were instantaneous observations, and these observations were easily affected by external stimuli. Therefore, they were considered less suitable as signal indicators.

The final question was to investigate whether this limited selection of five signal indicators had the same discriminative capacity as the set of 12 measurements. We determined the percentage of exceeded warning thresholds per indicator and calculated the sum of exceedances per farm for both the set of five and of 12 measurements. Table 4 gives an overview of these exceeded thresholds. The threshold level for the CO_2_ level was most often exceeded (38%), followed by the NH_3_ level (23%) and the thresholds for huddling (17%), ear score (13%), pen fouling (10%), and eye score (7%).

**Figure 2:**
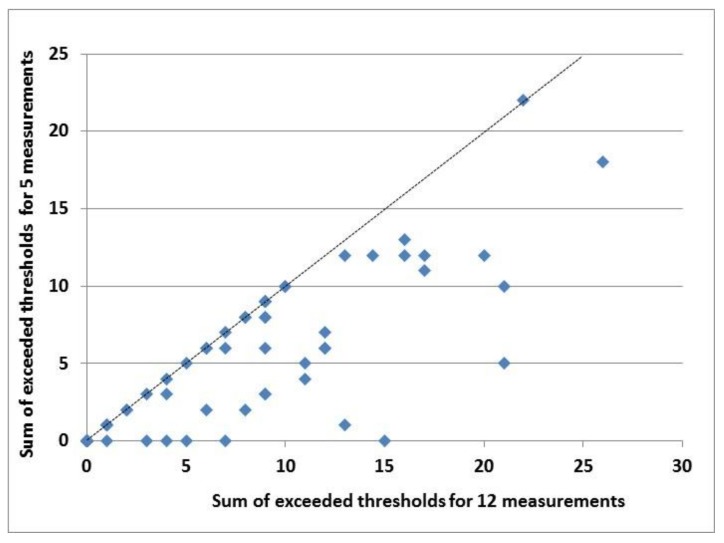
Relationship between sums of exceeded thresholds for five and for 12 measurements (r_s_ = 0.81; t-prob = 0.000) for farms (*n* = 64) with weaners; the dashed line indicates identical sums for both sets.

Farms were ranked based on their total number of violated thresholds. With a Spearman rank correlation between the two sums of violated thresholds in the datasets of five and 12 measurements in the weaner data, we found that the ranking of the farms was significantly correlated: r_s_ = 0.81; t-prob. = 0.000. This correlation is visualised in Figure 2.

### 3.2. Growing–Finishing Pigs

The data of the growing–finishing pigs from 32 farms are summarized at pen level in Table 5.

**Figure 3:**
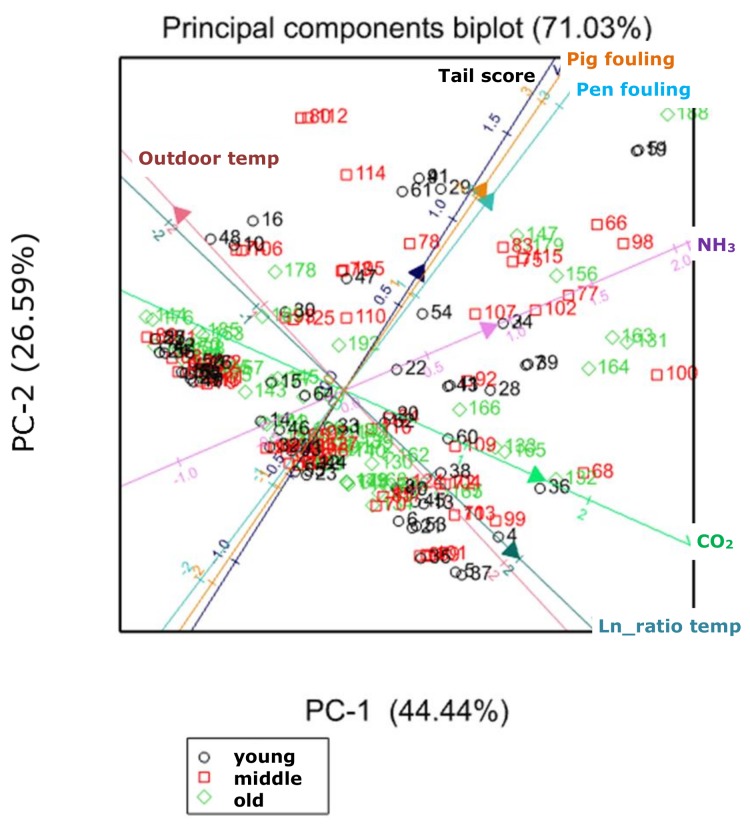
Basic biplot for growing–finishing pigs, including the outdoor temperature, natural logarithm (ln) of the ratio between the indoor and outdoor temperatures, NH_3_ and CO_2_ levels, pig fouling, and pen fouling, with tail score as an additional indicator.

The principal components analysis of the growing–finishing pig dataset resulted in a basic biplot with the CO_2_ and NH_3_ levels; pig fouling and pen fouling; outdoor temperature; and the ratio between outdoor and indoor temperature, as a measure of the ventilation rate. In Figure 3, this basic biplot is shown with the additional axis of tail score. The axes for pig and pen fouling were almost identical. Subsequently, the distinct pig-based indicators were compared with the basic axes in the biplot. Indicators support each other when their axes are pointing in the same direction. Eye and tail scores and panting were closely related to fouling. The behavioural indicators showed a weak relationship with the parameters in the basic biplot. Age group was a risk factor for NH_3_, with the highest NH_3_ level seen in the oldest group (aged 21–25 weeks).

Relationships between the NH_3_ and CO_2_ levels and the animal-based indicators were analysed with a mixed model regression analysis, and the relationships between the mutual animal-based indicators were analysed using cross-table analysis with Genstat [13]. Table 6 shows the significant relationships (*p* < 0.05) of the analysis of the most important growing–finishing pig measurements.

The results showed that the outdoor temperature was a risk factor for climatic conditions indoors; i.e., at low outdoor temperatures, we found reduced air quality (especially higher CO_2_ measurements), due to a lower ventilation rate. This means that at fixed upper limits for CO_2_ and NH_3_, the likelihood of an exceedance will be higher on colder days. The older pigs had significantly higher NH_3_ levels (*p* = 0.009). This means that this group is more at risk of violating the fixed threshold values for NH_3_. The measurements of pig fouling and pen fouling were significantly correlated (*p* < 0.01), therefore the use of only one of the two measurements is appropriate. All three animal characteristics of eye score, tail score, and panting were related to pen fouling. Tail score and panting were also significantly related. No relationship between the NH_3_ level and the animal characteristics of panting, pumping, and tail score was found. A significant relationship was found between the NH_3_ level and eye score, and a significant relationship was found between the NH_3_ level and pen fouling. CO_2_ level, huddling, and ear score were not related within this dataset, and the CO_2_ level had a significant relationship with the outdoor temperature and percentage of standing animals. Shivering comprised only two cases of score 1, and was therefore omitted from the statistical analyses.

The analysis resulted in a set of signal indicators with the environmental and animal-based measurements supporting each other. These were the CO_2_ level, NH_3_ level, tail score, eye score, and pig fouling. The CO_2_ and NH_3_ levels were indicators of air quality, and were both negatively related to the outdoor temperature. Pig and pen fouling were almost identical, so recording only pig fouling should be sufficient. Eye score, tail score, and panting were strongly related to fouling and the NH_3_ level. Coughing/sneezing and huddling were closely related to the CO_2_ level and outdoor temperature, and were difficult to measure. As in the measurements of the weaner pigs, the behavioural indicators within this project were instantaneous observations and were therefore easily affected by external stimuli. Therefore, they were considered as less suitable signal indicators.

**Figure 4:**
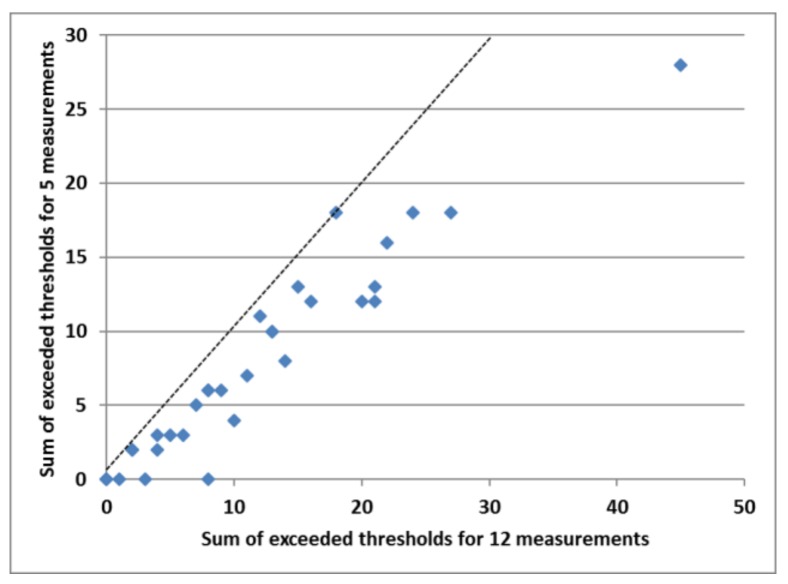
Relationship between sums of exceeded thresholds for five and for 12 measurements (r_s_ = 0.95; t-prob = 0.000) for farms (*n* = 32) with growing–finishing pigs; the dashed line indicates identical sums for both sets.

We then tested whether this reduced set of five signal indicators had the same discriminative capacity as the full set of 12 measurements. We determined the percentage of exceedances of the warning thresholds per indicator, and calculated the sum of the exceedances per farm both for the set of five and of 12 measurements. Table 7 gives an overview of the percentage in which the thresholds were violated for the growing–finishing pig farms. The level of CO_2_ most often exceeded the threshold value (33.3%), and threshold values were almost equally violated for NH_3_ levels, pig fouling, and pen fouling. Further violations were recorded for eye score (21%), huddling (16%), and ear score (10%). Farms were ranked based on the total number of threshold values that they had violated. With a Spearman rank correlation, we tested the relationship between the order of the farms based on the two sums of exceeded thresholds in the sets of five and 12 measurements, which was highly significant: r_s_ = 0.95; t-prob. = 0.000. This relation is visualised in Figure 4.

## 4. Discussion

This pilot study on the Dutch principle-based standards for the climate in pig houses has given clear pointers for how to identify farms with troublesome climatic conditions. Out of 22 measurements, 12 were suitable for inclusion, and from these, five were identified as the best signal indicators. Based on these five signal indicators, a first identification of problem farms could be made. The identified farms would require further investigation to prove if the principle-based standard for the indoor climate is met. However, such decisions are up to the competent authority. Even though the size of the dataset (96 farms) was limited, the unanimous results for farms with weaners or growing–finishing pigs confirm the validity of the detected signal indicators [18] and the potential of principle-based regulation [1].

A limitation of this study is that the farms were visited in a relatively cold period (average outdoor temperature < 15 °C). Low ventilation rates due to relatively low outdoor temperatures during the winter led to less replacement of air. This resulted in increased concentrations of CO_2_ and NH_3_ and relatively high room temperatures, which increase the risk of respiratory problems, such as those detected by Fablet et al. [12]. In the summer, pigs should be offered cooling facilities during periods of heat stress [14]. At body temperatures above the upper critical temperature, pigs cannot maintain homeostasis, resulting in impaired welfare and health, and ultimately resulting in death [15]. To what extent the detected signal indicators are also valid at high outdoor temperatures cannot be concluded from this study. Additional research for the best indicators of heat stress is necessary.

There is a higher risk of exceeded thresholds of CO_2_ and NH_3_ concentrations during winter. From January to March, more thresholds were exceeded than from April to June [18]. Although air quality should also be acceptable during winter, it might be useful to have seasonal signal indicators. 

The eye score was positively correlated with animal fouling and pen fouling, the NH_3_ level, and pigs lying isolated. As with weaner pigs, this is not surprising for growing–finishing pigs. Ammonia is a gas that irritates the eyes and is present in high concentrations in pens with high pen fouling scores. These findings reconfirm that eye score is a good animal-based indicator, as described by Telkänranta et al. [16].

The pig farms were only visited once, so no conclusion can be drawn about the repeatability of the measurements. In order to maximize repeatability within farms as well as within inspectors, it is important to have a strict protocol that is used consistently by thorough training of all inspectors, as proposed in the WQ pig protocol [10]. In our situation, we trained a team of ten inspectors who carried out the farms visits in pairs of changing composition. There was no examination at the end of the training. The aim was to minimize the variation in assessments and scores between inspectors. These mixed pairs guaranteed the exchange of experience and were preferred compared to using individual inspectors. However, periodical training of inspectors should be an essential part of the proposed signalling system.

The dataset included environmental measurements as well as animal-based measurements. However, space allowance and volume per pig did not have a relationship with any of the animal measurements. The Dutch legislation has clear minimum standards regarding the required surface area for pigs [2]. These environmental measurements showed little variation and were therefore excluded from the analysis. In contrast, room temperature and air quality were highly variable and could be adjusted with the ventilation system. However, air speed and draught are difficult to visualize and quantify during an inspection. Pigs do not like draught, as well as other environmental factors, and this can contribute to the development of abnormal behaviour, such as ear and tail biting [10,11,14]. In this project, the relationships between the environment-based measurements and animal-based measurements were stronger in the growing–finishing pigs than in the weaners. Technically speaking, a pig farmer has sufficient operational solutions to compensate for shortcomings. To solve problems with cold air or draught, a smoke test can help to detect air leakage, and holes can be sealed. At low temperatures, additional heating can be applied, and at high temperatures, water cooling systems can be used for cooling the pigs.

Several pairs of measurements were strongly correlated, and using only one of the two may suffice without the loss of information. This is the case, for example, for pen fouling and pig fouling. When choosing between an animal-based or environmental indicator, it can be preferable to assess the animal welfare from the animal itself [10,14]. However, the use of environmental measurements may better fit within the existing legislation as it is legally required for pigs to “have access to a clean and comfortable area with adequate drainage, where all pigs can lie at the same time” [2] (Article 2.16a).

After analysis, we found that the CO_2_ concentration, NH_3_ concentration and the three animal-based measurements of eye score, tail score, and ear score (for weaners) or fouling (for growing–finishing pigs) were the main signal indicators. The CO_2_ concentration, NH_3_ concentration, tail score, and eye score were good signal indicators for both the weaners and the growing–finishing pigs. Use of the CO_2_ and NH_3_ levels as signal indicators would align with poultry legislation and German legislation [3]. We identified pig fouling as the fifth signal indicator in growing–finishing pigs, with the ear score as the fifth indicator for weaners. This means that ear biting is a typical “weaner disorder” [11], and pig fouling is more common on a partly solid floor as is the case for growing–finishing pigs.

The effectiveness of inspections may also be increased by focusing further on risky situations. For example, the risk of exceeding the NH_3_ threshold was higher in older growing–finishing pigs. In addition to the selection of pens with signs of deficiencies, measurements could be restricted to the oldest animals.

Of the total set of indicators in the growing–finishing pigs and in the weaners, 12 were selected for further statistical analysis. Of this set of 12, only five measurements remained as signal indicators. Although the redundant measurements did not add much, they could be used as supporting evidence. This also applies to indicators on the farm level such as mortality rates, use of antibiotics, and slaughterhouse information [12]. However, they cannot always be linked to climatic problems because of their multifactorial origin and also because of the instantaneous sampling on-farm. For a reliable climate assessment of a farm, these measurements can still be relevant, even though the cause of high mortality cannot be solely linked to the indoor climate. Other factors are also responsible, so the conclusion of shortcomings in the indoor climate can only be drawn based on violated thresholds in multiple signal indicators.

For the pig farmer, the possibility of benchmarking is a highly relevant outcome of an inspection: “What is my position concerning indoor climate compared to my colleagues?” and: “Do the measures I have taken meet the legal requirements?”. The conclusion that the pig welfare does not comply with the legal standard always has to be based on actual exceeded thresholds of multiple factors, including animal-based measurements. High CO_2_ and NH_3_ levels alone, without any detectable negative consequences in the pigs, are not sound evidence of a suboptimal climate. After the first indication of a potential problem, more thorough investigation is required for better proving of a violation of the law. Often, the improvement of indoor climate is accompanied by better performance, as suggested in an EFSA study [10] and by slaughterhouse data [12]. Improving the indoor climate therefore provides a good perspective for the farmer in terms of profit, as well as for the animal in terms of improved animal welfare.

## 5. Conclusions

From this study, we can conclude that for detecting farms with climate problems, a comprehensive set of environmental and animal-based measurements can be reduced to a set of five measurements (signal indicators), almost without the loss of information. The ranking of farms based on the number of exceeded threshold values when using five or twelve signal indicators was almost equal.

The signal indicators for weaners were the NH_3_ and CO_2_ levels, ear score, eye score, and tail score. The signal indicators for growing–finishing pigs were the NH_3_ and CO_2_ levels, pig fouling, eye score, and tail score.

The data did not contain inspection days with high outdoor temperatures (>27 °C), and the suitability of the signal indicators at high outdoor temperatures is yet to be confirmed.

In the case of growing–finishing pigs, most problems with the indoor climate were observed in the oldest growing–finishing pigs (before slaughter). Focusing on these pigs seems to be a cost-effective measure when inspecting the indoor climate for pigs.

## Figures and Tables

**Table 1:** Registered measurements with threshold values for weaners and growing–finishing pigs.

Parameter	Threshold Values Weaners (7–25 kg)	Threshold Values Growing–Finishing Pigs (25–120 kg)	Source
Outdoor temperature (°C)	-	-	-
Room temperature (°C)	20–31 °C (LCT + UCT * at 20 kg)	13–23 °C (LCT + UCT * > 60 kg)	[15]
CO_2_ pig level (ppm)	3000	3000	[3]
NH_3_ pig level (ppm)	20	20	[3]
Pig fouling (0–2)	1	1	[10]
Pen fouling (0–2)	1	1	[10]
Eye score (0–4)	1	1	[16]
Tail score (0–2)	1	1	[10]
Ear score (0–2)	1	1	[10]
Panting (n)	-	-	[10]
Pumping (n)	-	-	[10]
Coughing/sneezing (n/5 min)	-	-	[10]
Huddling (0–2)	1	1	[10]
Shivering (0–2)	1	1	[10]
Lying isolated (0–2)	1	1	-
Posture (0–2)	1	1	-
Standing pigs (n)	-	-	-
Mortality incl. euthanasia (%)	>5%	>6%	-
Antibiotics (doses/pig/year)	>22	>10	[17]
Pleurisy (%)	N/A	>25%	-
Pneumonia (%)	N/A	>10%	-
Space allowance (m²/pig)	0.2	0.8	-
Room volume (m^3^/pig)	0.8	2.5	-

* LCT + UCT = lower and upper critical temperature; - = no limit value available; N/A = not applicable.

**Table 2:** Summary of climate- and behaviour-related measurements in pens of 64 farms with weaners.

Measurement	N Pens	Average	Median	Min	Max	Stdev
Pigs per pen	349	27.2	18	3	311	34.8
Pen length (m)	348	3.69	3.0	2	31	3.30
Pen width (m)	348	2.45	2.1	1	8	1.29
Pens per room	349	7.61	7	1	24	4.09
Room length (m)	347	11.4	11.0	4	26	4.82
Room width (m)	347	6.39	5.0	2	23	4.51
Room height (m)	349	2.83	3.0	1	6	0.73
Outdoor temperature (°C)	349	9.42	8.0	0	27	6.01
Indoor temperature (°C)	349	25.5	26.0	18	30	2.28
CO_2_ level (ppm)	349	2770	2700	34	6200	1.15
NH_3_ level (ppm)	346	14.4	10.0	1	68	11.7
Pig fouling score	349	0.09	0.0	0	2	0.36
Pen fouling score	349	0.12	0.0	0	2	0.37
Eye score	349	0.14	0.0	0	3	0.49
Tail score	349	0.04	0.0	0	2	0.22
Ear score	349	0.18	0.0	0	2	0.48
Panting	349	0.19	0.0	0	10	1.02
Pumping	349	0.14	0.0	0	5	0.66
Sneezing/coughing	349	4.13	0.0	0	150	15.5
Huddling	349	0.22	0.0	0	2	0.52
Shivering	349	0.02	0.0	0	1	0.14
Lying separately	349	0.15	0.0	0	2	0.40
Posture	349	0.63	0.0	0	2	0.71
Standing pigs (n)	349	24.7 (91%)	14.0	0	300	34.3
Mortality (%)	64	4.56	3.0	0	17	31.6
Antibiotics	64	9.76	5.0	0	50	22.0
Space allowance (m^2^/pig)	349	0.42	0.36	0.15	3.38	0.34
Room volume (m^3^/pig)	349	1.39	1.2	1.0	4.38	0.73

**Table 3:**
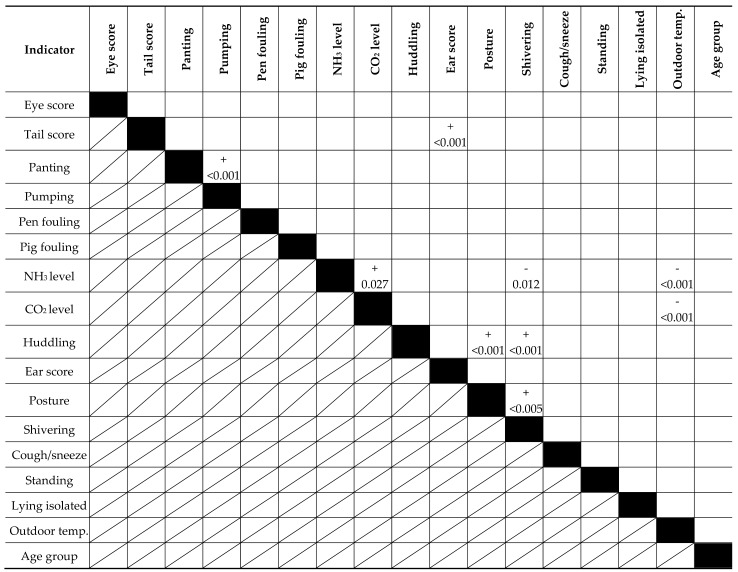
Relationships between measurements for weaners based on mixed model regression analysis or cross-table analysis (only shown when *p* < 0.05).

The “+” and “-“ indicate the direction of the relationship, with the value below showing the *p*-value.

**Table 4:** Full and reduced datasets for farms with weaners indicated with “X”, threshold values, and the percentage of farms that exceeded the threshold values.

Measurements	Full Dataset (*n* = 12)	Reduced Dataset (*n* = 5)	Threshold Value	Exceeded Thresholds
CO_2_ level	X	X	3000 ppm	38.8%
NH_3_ level	X	X	20 ppm	23.6%
Eye score	X	X	1	7.5%
Tail score	X	X	1	3.4%
Ear score	X	X	1	13.5%
Pig fouling	X		1	6.6%
Pen fouling	X		1	10.1%
Panting	X		0.2	2.3%
Pumping	X		0.2	1.4%
Coughing/sneezing	X		1	6.9%
Huddling	X		1	17.8%
Shivering	X		1	2.0%

**Table 5:** Summary of climate- and behaviour-related measurements in pens on 32 farms with growing–finishing pigs.

Measurement	N Pens	Average	Median	Min	Max	Stdev
Pigs per pen	183	13.1	12	3	50	7.69
Pen length (m)	182	3.99	3.87	1.98	8.7	1.26
Pen width (m)	182	2.58	2.4	1.53	6.71	0.84
Pens per room	188	12.1	12	1	40	8.09
Room length (m)	188	16.0	15	3	36	7.76
Room width (m)	188	9.15	8	3	23	3.79
Room height (m)	188	3.46	3	2	8	1.01
Outdoor temperature (°C)	32	11.5	9.5	1	27	7.03
Indoor temperature (°C)	184	23.8	24	17	28	2.66
CO_2_ level (ppm)	183	2425	2300	400	4700	109
NH_3_ level (ppm)	183	15.2	11	1	69	12.6
Pig fouling score	192	0.42	0	0	2	0.70
Pen fouling score	192	0.40	0	0	2	0.69
Eye score	192	0.30	0	0	3	0.67
Tail score	192	0.06	0	0	2	0.26
Ear score	192	0.12	0	0	2	0.39
Panting	192	0.20	0	0	6	0.85
Pumping	192	0.05	0	0	3	0.32
Sneezing/coughing	192	0.71	0	0	20	2.48
Huddling	192	0.20	0	0	2	0.49
Shivering	192	0.01	0	0	1	0.10
Lying separately	192	0.22	0	0	2	0.49
Posture	192	0.43	0	0	2	0.65
Standing pigs (n)	192	10.2	9	0	50	7.87
Space allowance (m^2^/pig)	178	0.87	0.82	0.30	1.74	0.24
Room volume (m^3^/pig)	184	3.75	3.39	0.75	10.88	1.64
Mortality (%)	32	6.03	2	0	123	21.2
Antibiotics (doses/pig/year)	32	1.88	1	0	17	3.12
Pleurisy (%)	32	6.19	2	0	46	10.9
Pneumonia (%)	32	2.44	1.5	0	12	3.13

**Table 6:**
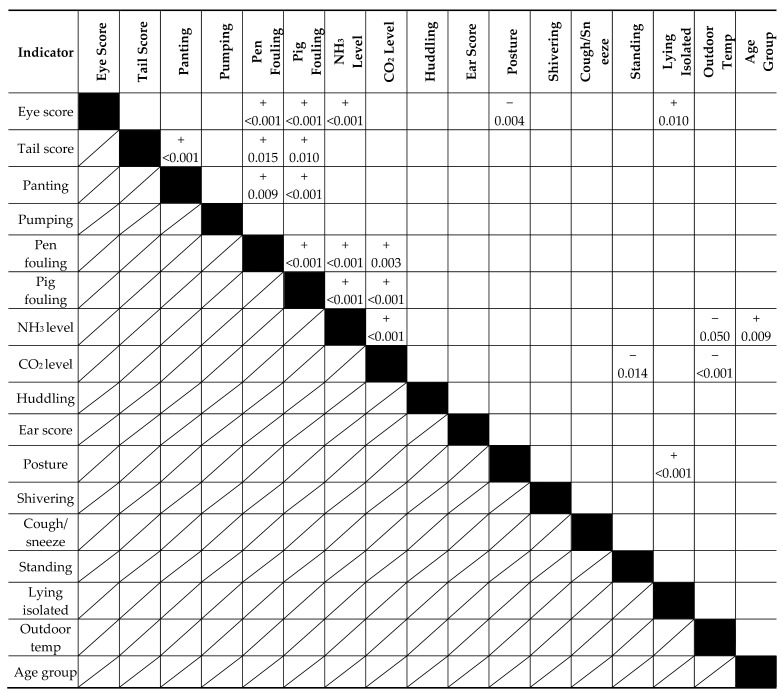
Relationships between measurements for growing–finishing pigs based on mixed model regression analysis or cross-table analysis (only shown when *p* < 0.05).

The “+” and “−“ indicate the direction of the relationship, with the value below showing the *p*-value.

**Table 7:** Full and reduced datasets for farms with growing–finishing pigs, threshold values, and the percentage of farms exceeding the threshold values.

Measurements	Full Dataset (*n* = 12)	Reduced Dataset (*n* = 5)	Threshold Value	Exceeded Thresholds
CO_2_ level	X	X	3000 ppm	33.3%
NH_3_ level	X	X	20 ppm	31.7%
Eye score	X	X	1	21.3%
Tail score	X	X	1	6.6%
Ear score	X		1	10.4%
Pig fouling	X	X	1	31.1%
Pen fouling	X		1	29.5%
Panting	X		0.2	5.5%
Pumping	X		0.2	0.5%
Coughing/sneezing	X		1	1.1%
Huddling	X		1	16.4%
Shivering	X		1	1.1%
